# Robust Production of Merkel Cell Polyomavirus Oncogene Specific T Cells From Healthy Donors for Adoptive Transfer

**DOI:** 10.3389/fimmu.2020.592721

**Published:** 2020-12-09

**Authors:** Sarah I. Davies, John Barrett, Susan Wong, Mark Jesse Chang, Pawel J. Muranski, Isaac Brownell

**Affiliations:** ^1^ Hematology Branch, National Heart, Lung, and Blood Institute, Bethesda, MD, United States; ^2^ Department of Microbiology & Immunology, Georgetown University Medical Center, Washington, DC, United States; ^3^ Dermatology Branch, National Institute of Arthritis and Musculoskeletal and Skin Diseases, National Institutes of Health (NIH), Bethesda, MD, United States; ^4^ Columbia Center for Translational Immunology (CCTI), Cellular Immunotherapy Laboratory, Columbia University Medical Center, New York City, NY, United States

**Keywords:** CD4^+^ T-lymphocytes, skin neoplasms, immunotherapy, adoptive, translational medial research, cellular immunity, donor, allogeneic

## Abstract

Virus positive Merkel cell carcinoma (VP-MCC) is an aggressive but immunogenic skin malignancy driven by Merkel cell polyomavirus (MCPyV) T antigen (TAg). Since adoptive T cell transfer (ACT) can be effective against virus-driven malignancies, we set out to develop a methodology for generating MCPyV TAg specific T cells. MCPyV is a common, asymptomatic infection and virus-exposed healthy donors represent a potential source of MCPyV TAg specific T cells for ACT. Virus specific T cells were generated using monocyte-derived dendritic cells (moDCs) pulsed with MCPyV TAg peptide libraries and co-cultured with autologous T cells in supplemented with pro-inflammatory and homeostatic cytokines for 14 days. Specific reactivity was observed predominantly within the CD4^+^ T cell compartment in the cultures generated from 21/46 random healthy donors. Notably, responses were more often seen in donors aged 50 years and older. TAg specific CD4^+^ T cells specifically secreted Th1 cytokines and upregulated CD137 upon challenge with MCPyV TAg peptide libraries and autologous transduced antigen presenting cells. Expanded T cells from healthy donors recognized epitopes of both TAg splice variants found in VP-MCC tumors, and minimally expressed exhaustion markers. Our data show that MCPyV specific T cells can be expanded from healthy donors using methods appropriate for the manufacture of clinical grade ACT products.

## Introduction

Virus positive Merkel cell carcinoma (VP-MCC) is an aggressive neuroendocrine skin cancer mediated by clonally integrated Merkel cell polyomavirus (MCPyV) (1). Immune checkpoint inhibitors (ICIs) produce frequent and durable responses in patients with advanced VP-MCC (2–4), highlighting a critical role for T cell immunity in tumor clearance. Therefore, enhancing anti-tumor immunity with adoptive T cell transfer (ACT) of tumor-specific T cells could improve responses to ICIs and offer hope to patients where ICIs are contraindicated or who are refractory to ICIs alone (NCT02584829, NCT03747484) (5, 6). Current clinical trials that use VP-MCC tumor specific T cells are human leukocyte antigen (HLA)-restricted and are only applicable to a subset of VP-MCC patients (NCT02584829; NCT03935893; NCT03747484). By using overlapping peptide libraries and enhanced culture conditions, we found that healthy donor peripheral blood can be used to reliably manufacture VP-MCC specific T cells suitable for allogeneic ACT regardless of HLA genotype.

**Figure 1:**
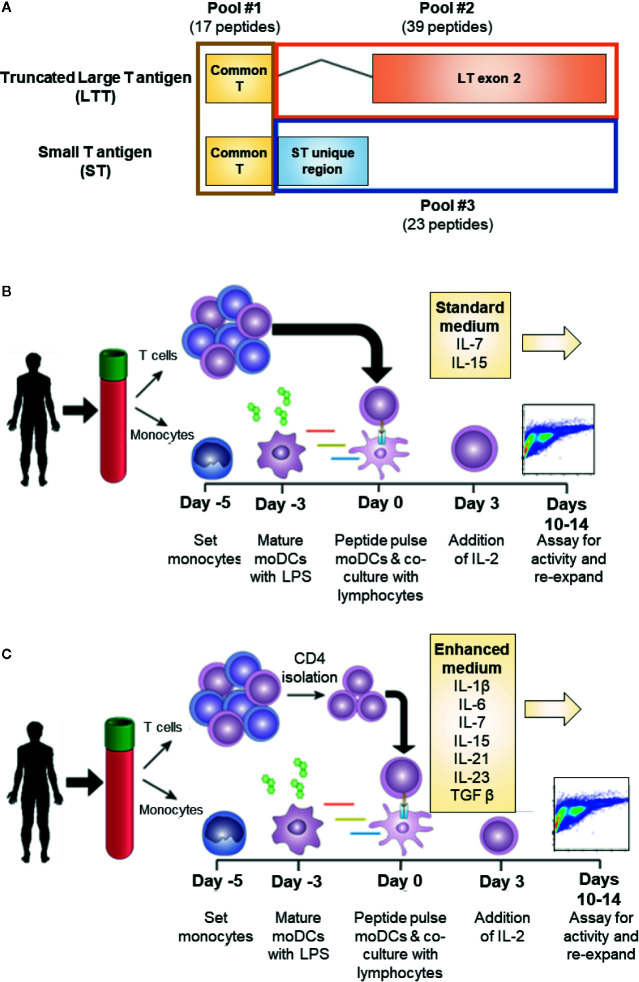
Experimental conditions for MCPyV T antigen specific T cell expansion. **(A)** Schema of MCPyV T antigen regions represented in overlapping peptide library pools used in this study. Peptides were 15 amino acids long with an 11 amino acid overlap. **(B)** Standard *in vitro* antigen specific T cell expansion culture conditions. Briefly, monocyte derived dendritic cells from elutriated monocytes are peptide pulsed then co-cultured with autologous elutriated lymphocytes in the presence of IL-7 and IL-15. Seventy-two hours later, IL-2 is added to the culture medium. Cells are cultured for 10-14 days then challenged with peptide prior to analysis. **(C)** Enhanced *in vitro* antigen specific T cell expansion culture conditions. Peptide-pulsed monocyte derived dendritic cells are co-cultured with autologous CD4^+^ T cells and cultured in the presence of IL-1β, IL-6, IL-7, IL-15, IL-21, IL-23, and TGFβ. IL-2 is added 72 h later. Cells are cultured for 10–14 days then challenged with peptide prior to analysis.

MCPyV is a small, non-enveloped double-stranded DNA virus that typically causes an asymptomatic infection in most individuals during infancy (7). The pathogenesis of VP-MCC requires the clonal integration of MCPyV into the host genome in addition to a large truncating mutation of the T antigen (TAg) gene. MCPyV TAg is a major oncogene of VP-MCC and is expressed homogenously in all VP-MCC tumor cells (8). In VP-MCC, TAg is expressed as two alternatively spliced products: small T antigen (ST) and truncated large T antigen (LTT; **Figure 1A**) (9, 10). Both proteins are required for the proliferation and survival of VP-MCC cells (8). Addiction of VP-MCC cells to MCPyV oncoproteins makes TAg an attractive target for immunotherapy. Moreover, the somatic mutational burden of VP-MCC is extremely low, suggesting the tumors produce few additional neoantigens (11).

T cell immunity directed against MCPyV TAg is associated with longer survival and fewer metastases in VP-MCC patients (12). ACT of autologous CD8^+^ MHC class I restricted T cells specific for a HLA-A*24:02-restricted MCPyV LT-Ag_92-101_ TAg epitope mediated regressions of VP-MCC (13) and this tetramer approach is now in early clinical trials to treat VP-MCC (NCT01758458). HLA-A02-restricted engineered TCR is also in phase I/II clinical trials (NCT03747484). However, the universal applicability of this approach is hindered by its HLA-restricted nature, which limits patient eligibility and also risks relapse from HLA down regulation by the tumor (14). Furthermore, relying on autologous cells might exclude many patients with VP-MCC who have dysfunctional T cells, are lymphopenic, or are immunocompromised.

An alternative immunotherapeutic strategy would be to transfer allogeneic MCPyV-specific T cells derived from an HLA matched healthy donor using peptide libraries of the viral oncoproteins. ACT with peptide library-pulsed *ex vivo* expanded virus or tumor antigen-specific T cells has been used with success in multiple clinical trials to treat viral infections, EBV-driven lymphomas, and solid tumors (15–17). MCPyV TAg specific CD4^+^, but not CD8^+^, T cells have been found in healthy donors and have not been evaluated for ACT (12, 18–20). Here, we show that polyclonal MCPyV TAg specific CD4^+^T cells appropriate for ACT treatment of patients with VP-MCC can be generated *in vitro* from healthy donors using methods easily adaptable to clinical grade manufacture.

## Materials and Methods

### Sample Acquisition

Elutriated peripheral monocytes and lymphocytes from healthy donors were obtained from the Department of Transfusion Medicine, NIH, Bethesda, MD, USA under NIH IRB-approved protocol 99-CC-0168 (NCT00001846). Research blood donors provided written informed consent and blood samples were de-identified prior to distribution. Cells were assessed for viability using trypan blue exclusion and cryopreserved in 10% DMSO (Sigma Aldrich), 20% human serum (Gemini Bio-Products), and 0.065 mg/ml gentamycin (Quality Biological) in RPMI (Gibco) for later use. HLA class I and class II sequence-based typing was performed by the NIH Department of Transfusion Medicine’s HLA Laboratory.

### Culture Media

Primary leukocytes were cultured in cell growth medium in the presence of indicated cytokines. Cell growth medium contains 45% RPMI 1640, 50% AIM V CTS (Gibco by Life Technologies), and 5% non-AB pooled human serum (Gemini Bio-Products) by volume and was supplemented with 65 ug/ml gentamycin (Quality Biological), and 1X penicillin-streptomycin-glutamine solution (Gibco by Life Technologies).

### Generation of Monocyte-Derived Dendritic Cells

Elutriated monocytes were thawed and plated in a 6 well plate at up to 1 × 10^7^ cells/well in cell growth medium supplemented with 1,000 IU/ml recombinant human (rh) GM-CSF and 1,000 IU/ml rhIL-4 (Peprotech) for 72 h. Monocyte-derived dendritic cells (moDCs) were matured with 1.25ug/ml lipopolysaccaride (*Escherichia coli* O127:B8; Sigma), 1,000 IU/ml rhGM-CSF, and 1,000 IU/ml rhIL-4 for 48 h. Harvested monocyte derived dendritic cells cell density and viability were determined by trypan blue exclusion.

### Primary moDC Transduction

On day four of moDC culture, cells were plated at 4 × 10^5^ cells/well in a 96 well plate. Cells were transduced using recombinant lentiviral vectors encoding codon optimized genes for ST, LTT, GFP, and MAGE-A3 single infections. Cells were infected at a MOI of 20 and spinoculated at 1800xG at 33C for 2.5 h. Cells were then washed in warm medium, incubated overnight, and co-cultured with autologous expanded T cells. Intracellular cytokine analysis was performed as described above. Aliquots of each donor’s transduced cells were assayed by RT-PCR for construct expression. MAGE-A3, ST, and LTT codon optimized lentiviral construct (Cellomics, Pittsburgh, PA, USA) and PCR primer sequences are available in the **Supplementary Materials and Methods**.

### Peptide Libraries

Overlapping peptide libraries were used to activate antigen specific T cells. Each library contained overlapping peptides with a length of 15 amino acids and an overlap of 11 amino acids across the full-length of the protein sequence. MCPyV T antigen peptide libraries were based on the protein sequences LTT (Ref Seq: YP_009111421.1 truncated at amino acid 246) and ST (Ref Seq: YP_009111422.1). MCPyV peptide libraries were manufactured by Neobiolabs at >70% purity by HPLC. To investigate the antigenic contribution of each T antigen exon, the peptides libraries were divided into three pools: common region (amino acid 1–80; total 17 peptides), LTT exon 2 (LTT amino acid 80–246; total 39 peptides), and the unique ST (ST amino acid 80–186; total 23 peptides) region (**Figure 1A**). Irrelevant antigenic overlapping peptide libraries for WT1 and NY-ESO1 (JPT Peptide Technologies) were used as specificity controls where indicated. Amino acid sequences for each peptide used in this study can be found in the **Supplementary Materials and Methods**. Peptide libraries were suspended in DMSO at a working concentration of 250 ng/ul, and moDCs were pulsed at a final concentration of 1 ug/ml 1–2 h prior to culture.

### Generation of T Cell Lines

Antigen specific T cells were generated by stimulation of the lymphocyte population with peptide pulsed moDCs at a T cell to antigen presenting cell (APC) ratio of 5:1. Where indicated, starting lymphocyte populations were enriched for CD4^+^ T cells by immunomagnetic negative selection (EasySep Stem Cell Technologies). Cells were co-cultured in cell growth medium supplemented with either standard T cell growth cytokines of 10 ng/ml rhIL-7 and rhIL-15 (Peprotech) or enhanced cytokine conditions of 20 ng/ml rhIL-1β, 20 ng/ml rhIL-6, 10 ng/ml rhIL-7, 10 ng/ml rhIL-15, 50 ng/ml rhIL-21, 25 ng/ml rhIL-23, and 5 ng/ml rhTGFβ (Peprotech). After 72 h of culture, 30 IU/ml rhIL-2 was added to the culture. Cultures were fed or split every 2–3 days as needed. Cultures were re-stimulated up to 2 times with peptide pulsed moDCs every 10–14 days.

### T Cell Stimulation Assay

T cell lines were plated at 5 × 10^5^ cells per well in a 96 well plate and stimulated for 12 h either with anti-CD28 and CD49d antibodies (BD Biosciences) and peptide libraries, peptide pulsed autologous moDCs, or transduced moDCs. An effector to APC ratio of 7:1 was used for moDC re-stimulations. After 1 h, Golgi inhibitors brefeldin A and monensin (GolgiStop™ and GolgiPlug™; BD Biosciences) were added according to manufacturer’s instructions. An antigen-specific response was defined as secretion of IFNγ or TNFα by >0.5% of lymphocytes and a frequency of reactive cells at least twice that of negative controls. The CD137 expression was determined by flow cytometry 16 h post antigen stimulation in the absence of Golgi inhibitors. Negative controls were either irrelevant antigen or vehicle where indicated. A “positive donor” was defined as a donor whose expanded cells had at least two successful antigen-specific T cell responses to MCPyV T antigen.

### Flow Cytometry

Cells were plated in a 96 well plate at 0.5 × 10^6^ cells/well and washed with PBS. Between each wash, plated cells were centrifuged at 500xG for 5 min. Cells were washed again with PBS then stained with fixable viability stain (Invitrogen L34955) in a 96 well plate at 4°C for 30<nbsp/>min. Cells were washed with FACS buffer (2% FBS and 2 uM EDTA in PBS) then stained using the appropriate surface antibodies in FACS buffer for 30 min at room temperature. Cells were washed, permeabilized, and intracellularly stained using either the fixation/permeabilization solution kit (BD Biosciences) kit for cytokine targets or the FOXP3 transcription factor staining buffer set (eBiosciences) for transcription factor targets following respective manufacturer’s instructions. Samples were suspended and run on a BD Fortessa flow cytometer using an automated plate reader. Flow cytometry data were analyzed using FlowJo software (Treestar, Ashland, OR, USA). Gating strategies available in the **Supplementary Materials and Methods**. Polyfunctionality analysis was performed using SPICE v6 software (21).

### Flow Cytometry Antibodies

A list of antibodies for flow cytometry is available in the **Supplementary Materials and Methods**.

### Statistics

Statistics were performed using GraphPad Prism v7. Average and standard error of the mean are displayed in bar charts.

## Results

### MCPyV TAg Specific T Cells are Efficiently Generated from Healthy Donor Blood

**Figure 2:**
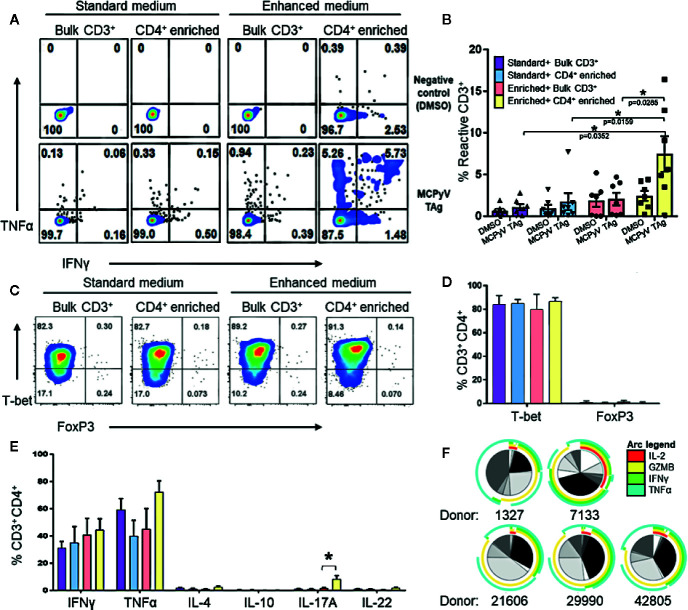
Expansion of MCPyV T antigen specific T cells is improved by CD4^+^ enrichment and culture in enhanced cytokine cocktail. Donor T cells were expanded for 2 weeks in ^+^the following cytokine and cell enrichment conditions: standard cytokines and bulk CD3^+^ T cells (purple), standard cytokines and CD4^+^ enriched T cells (blue), enhanced cytokines and bulk CD3^+^ T cells (orange), and enhanced cytokines and CD4^+^ enriched T cells (yellow). Frequency of IFNγ and TNFα-expressing CD3^+^ cells generated from **(A)** a representative donor and **(B)** seven paired donors after challenge with either MCPyV TAg peptides or vehicle control. **(C)** Frequency of T-bet and FOXP3 expressing CD3^+^CD4^+^ MCPyV TAg T cells from a representative donor after 2 weeks of culture in each condition. **(D)** Frequency of T-bet and FOXP3 expressing CD3^+^CD4^+^ MCPyV TAg T cells after 2 weeks of culture in each condition (n = 5 donors). **(E)** Frequency of Th signature cytokines IFNγ, TNFα, IL-4, IL-10, IL-17A, and IL-22 by live CD3^+^CD4^+^ cells from five donors after 2 weeks of culture in each condition followed by PMA-ionomycin stimulation. **(F)** SPICE plots depicting polyfunctional expression of IL-2 (red arc), Granzyme B (yellow arc), IFNγ (green arc), and TNFα (cyan arc) of CD3^+^CD4^+^TNFα^+^ cells from five donors stimulated by MCPyV peptides. Each wedge grayscale tone represents the frequency of T cells that express a unique combination of cytokines, as indicated by the colored arcs. Gating strategies are shown in **Supplementary Materials and Methods**. Standard error mean and significance from paired T-test are displayed. * = p value ≤ 0.05.

We investigated the role of various culturing conditions to improve the production of MCPyV TAg specific cells *ex vivo.* Cells from healthy donor blood was processed to generate moDCs. These cells were pulsed with TAg overlapping peptide libraries and co-cultured with autologous lymphocytes, allowing for the activation and expansion of reactive T cells. We initially used standard clinical production strategies to expand antigen-specific T cells, whereby lymphocytes were cultured with the cytokines IL-2, IL-7, and IL-15 as depicted in **Figure 1B** ((22–26); NCT02231853; NCT02694783). Standard cytokine containing medium did not yield detectable TAg specific cells. However, after enriching for CD4^+^ lymphocytes, standard cytokine medium achieved successful expansion in 2/12 tested donors (**Supplementary Figure 1**). Testing combinations of various cytokines in T cell culture media revealed that tumor antigen specific CD4^+^ T cell growth was improved by culturing in IL-1β, IL-2, IL-6, IL-7, IL-15, IL-21, IL-23, and TGFβ, and that the addition of TGFβ was critical for the improved performance of the cocktail (27). A manuscript characterizing the cytokine cocktail is in preparation. Applying this “enhanced” cytokine cocktail to CD4^+^ enriched cultures as depicted in **Figure 1C** lead to more frequent detection of expanded MCPyV TAg specific CD4^+^ T cells (**Figure 2B**). Using these conditions, MCPyV TAg specific T cells were generated from 21/46 (45.6%) random healthy donors.

### Expanded T Cells are Th1 Phenotype

To evaluate T cell polarization (Th) of the CD4^+^ T cells generated in the enhanced versus standard conditions, the expression of specific transcription factors and Th defining cytokines in stimulated cells from five donors were evaluated by flow cytometry. T cell cultures stimulated with overlapping peptide libraries and co-stimulated with anti-CD28 and anti-CD49d demonstrated that CD4^+^ enrichment resulted in the highest yield of TNFα and IFNγ-secreting T cells in response to cognate antigen (**Figures 2A, B**). Because cytokine exposure *in vitro* can encourage growth of different T cell polarizations, we validated that the cultured CD4^+^ phenotypes were associated with tumor rejection. We were especially interested in confirming absence of immunosuppressive Tregs that may inhibit anti-tumor efficacy and are promoted by TGFβ. As expected, 2 weeks of culture in the standard cytokines produced Th1 polarized CD4 T cells (22, 24–26). Despite the presence of TGFβ, culture in the enhanced cytokines also produced almost exclusively T-bet^+^FOXP3^-^CD4^+^ cells, suggesting a Th1 polarization (**Figure 2C, D**). Consistent with this, activating the cultured cells with PMA-ionomycin predominately induced expression of the Th1 cytokines TNFα and IFNγ in CD4^+^ T cells. CD4^+^ enriched cells grown in the enhanced cytokine conditions also produced a statistically significant increase of IL-17A-producing CD4^+^ T cells (**Figure 2E**). Additionally, MCPyV TAg-specific cells gated on CD3^+^CD4^+^TNFα^+^ cells were polyfunctional for IL-2 and the Th1 effector molecules granzyme B, IFNγ, and TNFα (**Figure 2F**), a feature associated with immune-mediated tumor clearance (28, 29). Cytotoxicity assays demonstrated that expanded MCPyV TAg-specific T cells do not induce killing of peptide pulsed autologous moDCs or phytohemagglutinin induced blasts (data not shown).

### MCPyV TAg Peptide-Expanded T Cells Specifically Recognize TAg Presented by Autologous APCs

**Figure 3:**
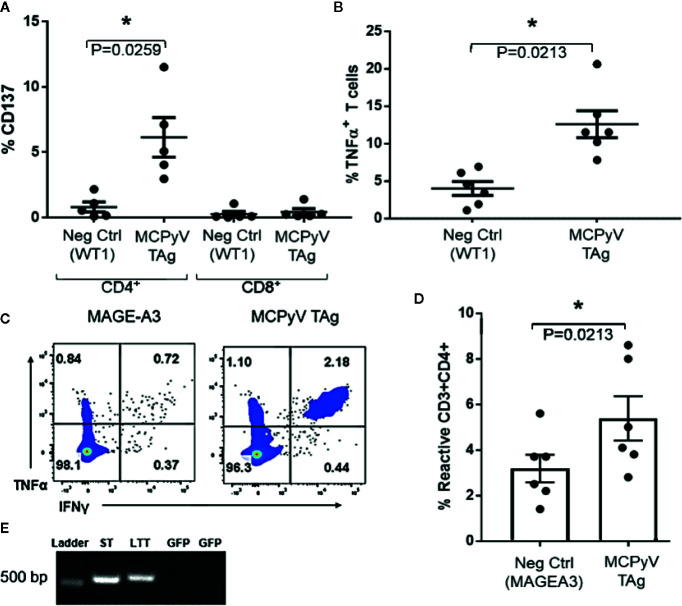
Expanded T cells specifically recognize MCPyV TAg presented by autologous moDCs. MCPyV TAg T cell cultures from positive donors were stimulated with moDCs peptide pulsed with either MCPyV TAg or WT1 libraries then assayed for the frequency of **(A)** CD137 (n = 5, p = 0.02) and **(B)** TNFα cytokine expressing (n = 6; p = 0.02) T cells. Expanded MCPyV T cell cultures from positive donors were stimulated with moDCs transduced with codon optimized MCPyV LTT, ST, or irrelevant MAGE-A3 constructs from **(C)** a representative donor and **(D)** the six paired donors. **(E)** Construct expression of moDCs transduced with either ST or LTT confirmed by RT-PCR. GFP specific primers were used as a negative control. Full gel picture and flow cytometry gating strategies are shown in **Supplementary Materials and Methods**. Standard error mean and significance from paired T-test are displayed. * = p value ≤ 0.05.

To investigate the specificities of the expanded T cells, we challenged MCPyV TAg reactive lines with antigen-loaded autologous moDCs. CD4^+^ T cell lines challenged with peptide-loaded autologous moDCs expressed the polarization independent T cell activation marker CD137 (n = 5; paired t test p = 0.02; **Figure 3A**) and the Th1 cytokine TNFα (n = 6; paired t test p = 0.02; **Figure 3B**) specifically in response to MCPyV TAg peptide relative to irrelevant peptide libraries. To confirm that peptide expanded T cells responded to naturally expressed epitopes from natively processed antigen, we challenged six MCPyV TAg expanded lines with TAg transduced autologous moDCs (**Figure 3E**). The greatest response to either ST or LTT construct is shown. Stimulation with transduced TAg-expressing moDCs consistently produced more reactive cells than stimulation with moDCs transduced with the irrelevant antigen MAGE-A3 (Paired t test p = 0.02; **Figures 3C, D**).

### All TAg Regions Expressed by VP-MCC Tumors are Immunogenic to Healthy Donors

**Figure 4:**
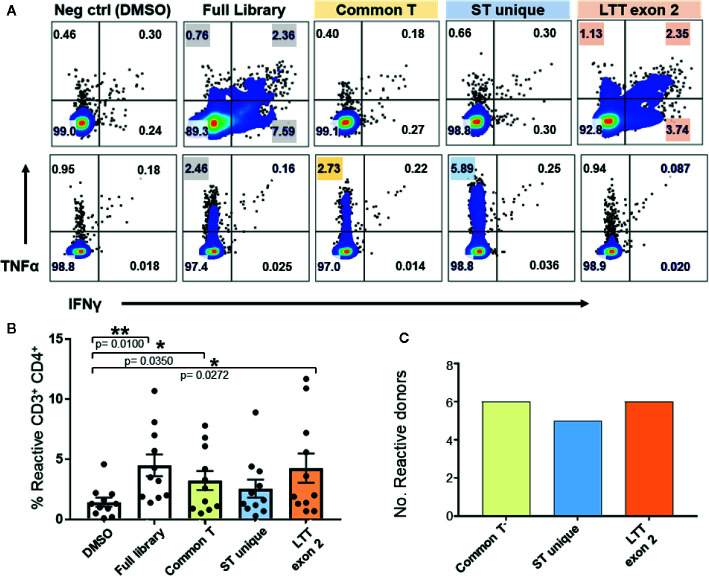
Expanded MCPyV TAg T cells from healthy donors recognize multiple epitopes of T antigen. Expanded MCPyV T cells from positive donors were challenged with peptide pools derived from the full TAg library, common T (yellow), ST unique (blue), and LTT exon 2 (orange) regions of TAg. **(A)** Representative donors’ reactivity to individual peptide pools demonstrating a monoclonal (top) and polyclonal (bottom) response. Reactive samples are highlighted. **(B)** Frequency of TNFα or IFNγ expressing CD3^+^CD4^+^ T cells in response to each peptide pool (n = 11). Standard error mean and significance from paired T-test are displayed. * = p value ≤ 0.05. ** = p value ≤ 0.01 **(C)** Number of assayed positive donors who specifically responded to each MCPyV TAg region (n = 10).

To identify the relative immunogenicity of MCPyV TAg protein domains in healthy donors, we stimulated TAg specific T cells with three separate TAg peptide pools (**Figures 1A** and **4A**). MCPyV TAg T cell lines from 12 donors all recognized at least one peptide pool, and 3/12 donors demonstrated a polyclonal response (**Figure 4C**). All three TAg regions were immunogenic among positive donors (**Figure 4B**). These data suggest that MCPyV TAg is rich in potential epitopes that a diverse healthy donor T cell repertoire can recognize.

### Expanded CD4+ MCPyV TAg T Cells Lack Terminal Differentiation and are Not Exhausted

**Figure 5:**
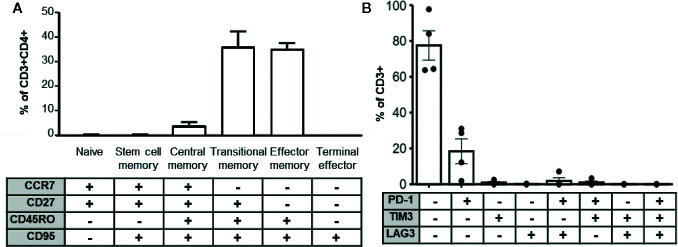
MCPyV TAg specific T cells possess clinically relevant phenotypes. **(A)** MCPyV TAg expanded CD3^+^CD4^+^ T cells assayed for differentiation status based on memory markers CCR7, CD27, CD45RO, and CD95 by flow cytometry (n = 6). Percentage of naïve (CCR7^+^CD27^+^CD45RO^−^CD95^−^), stem cell memory (CCR7^+^CD27^+^CD45RO^−^CD95^+^), central memory (CCR7^+^CD27^+^CD45RO^+^CD95^+^), transitional memory (CCR7^−^CD27^+^CD45RO^+^CD95^+^), effector memory(CCR7^−^CD27^−^CD45RO^+^CD95^+^), and terminal effector (CCR7^−^CD27^−^CD45RO^−^CD95^+^) T cells are shown. **(B)** Expression of canonical exhaustion markers PD-1, TIM3, and LAG3 by expanded CD3^+^ cells (n = 4). Gating strategies shown in **Supplementary Materials and Methods**.

Longevity and potency of adoptively transferred T cell products post-transplant is likely a function of immune memory formation and lack of exhaustion. Several studies also indicate that less differentiated T cell products are associated with superior performance *in vivo* (30–32). To determine whether the enhanced cytokine cocktail produced terminal effector cells, we determined the memory phenotype of expanded MCPyV TAg cells by the differential expression of the markers CCR7, CD27, CD45RO, and CD95 (33, 34). We also assayed exhaustion marker expression of the expanded T cell cultures. On average, CD3^+^CD4^+^ gated expanded cell cultures contained 4.03^+^/− 1.27% central memory, 36.2 ^+^/− 1.27% transitional memory, and 35.2 ^+^/− 5.66% effector memory cells (**Figure 5A**). The majority of cultured CD3^+^ cells did not express the canonical exhaustion markers PD-1, TIM3, or LAG3, but 18.4 ^+^/− 6.92% expressed PD-1 alone and 0.955 ^+^/− 0.567% expressed TIM3 alone. A minority of cells co-expressed LAG3^+^PD-1^+^ (1.87 ^+^/− 1.75%) or TIM3^+^PD-1^+^ (0.999 ^+^/− 0.775%). Frequency of other exhaustion marker co-expression combinations was <0.5% and no cells expressed all three exhaustion markers (**Figure 5B**). Taken together, these data demonstrate that the majority of expanded CD4^+^ MCPyV specific cells are not terminally differentiated and lack exhaustion markers, suggesting they would be suitable for ACT.

### Successful Expansion of MCPyV TAg Specific Cells is Associated with Advanced Donor Age

**Figure 6:**
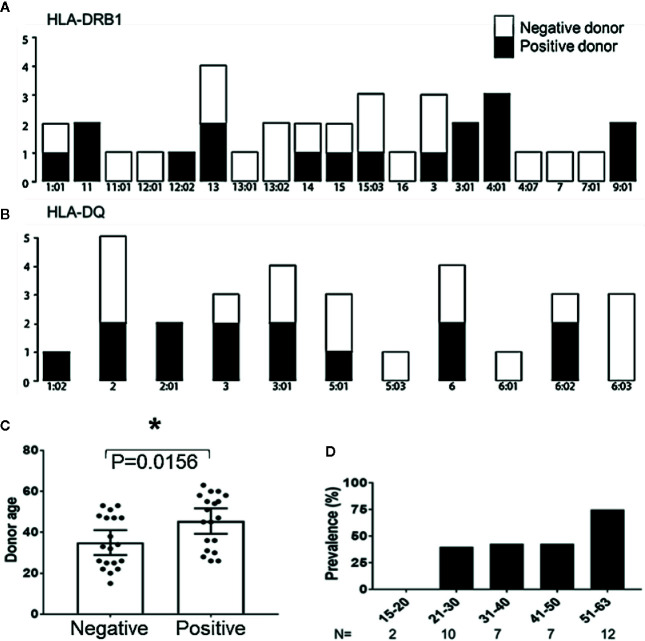
Age correlates with recovery of MCPyV TAg specific T cells. **(A)** HLA-DRB1 and **(B)** HLA-DQ alleles of positive and negative donors (n = 20; Fishers exact test > 0.1). **(C)** Positive and negative donor status by age (n = 38; p = 0.01). Significance determined by Student’s T-test. * = p value ≤ 0.05. **(D)** Prevalence of positive donors by age group.

To determine if certain donor HLA alleles were associated with successful expansion of peptide derived MCPyV TAg specific T cells, 20 donors used in this study were HLA typed and analyzed. Donors who produced MCPyV TAg reactive T cells in at least two independent assays were classified as positive responders. TAg specific cells were produced with no bias for donor HLA-A, B, Cw, DR, or DQ alleles (Fisher’s exact test p > 0.1 **Figures 6A, B**; **Supplementary Figure 2**). We then looked at whether additional donor characteristics such as age, sex, and race correlated with responder status. Of the 46 donors studied, 6 non-responder and 2 responders did not have donor age data available. Of the 38 evaluable donors, responders were older (median age 45.5 ^+^/− 2.97 years) compared to non-responders (34.9 ^+^/− 2.89 years); paired t test p = 0.01) (**Figure 6C**). Notably, 75% of donors over 50 years old were responders (n = 12; **Figure 6D**). No significant trends in sex or race were identified.

## Discussion

We have developed a novel method to rapidly and reliably expand healthy donor T cells *ex vivo* targeting the primary oncogene of VP-MCC using a GMP-adaptable methodology. We produced MCPyV TAg specific T cells with phenotypic characteristics favorable for clinical cell products. Cells were polyfunctional CD4^+^ memory T cells minimally expressing PD-1. Peptide-expanded TAg specific T cells responded to natively processed MCPyV antigen and not to irrelevant antigens presented by autologous APCs, demonstrating specific on-target activation. Furthermore, multiple epitopes within the two TAg gene products were immunogenic in HLA-unrestricted healthy donors, demonstrating a broad diversity of tumor relevant epitopes for VP-MCC immunotherapy.

Overlapping peptide libraries can recapitulate potential epitopes for an antigen of interest without the inefficiency of using whole protein and lack the clinical safety concerns of viral transfected APCs (35). However, synthetic peptides may produce an epitope not readily presented in a native context. Thus, it is critical to validate that peptide reactive cells can also recognize naturally processed antigens. Our TAg overlapping peptide libraries produced T cells that recognized immunogenic epitopes presented across various HLA alleles, and we confirmed that these peptide-generated T cells readily recognize naturally processed antigens presented by transduced autologous APCs. These data strongly suggest that the TAg peptide libraries accurately represent clinically relevant epitopes.

Our approach readily generated TAg specific CD4^+^ T cells from healthy donors that could be used in ACT. Although tumor eradication has classically been attributed to CD8^+^ cytotoxic T cells, it is increasingly recognized that CD4^+^T cells also play an important role in tumor control. Clinical trials have demonstrated that ACT and tumor infiltration by Th1 CD4^+^ T cells can mediate regression of advanced solid tumors (36–40). Recently it was shown that MCPyV TAg-specific CD4^+^ T cells are enriched in VP-MCC tumors (20), demonstrating that MCPyV MHC class II restricted TAg epitopes are relevant to tumor immunity. Th1 CD4^+^cells engage in canonical activation and recruitment of innate immune cells and CD8^+^ cells to direct anti-tumor immunity (41, 42). They also directly kill MHC class II bearing tumor or stromal cells and rescue ICI refractory anti-tumor CD8^+^ cells *in vivo* to clear tumors (43, 44). Murine models also demonstrated Th17 CD4^+^cells are capable of tumor eradication (45, 46). Lastly, since VP-MCC immune evasion to MCPyV TAg specific CD8^+^ can occur in VP-MCC tumors treated with checkpoint inhibitors and HLA class I restricted transduced TCRs (14), the ACT of VP-MCC-specific CD4^+^ T cell could be beneficial in reversing such evasion.

The hallmarks of successful T cell products for ACT are T cell persistence, polyfunctionality, and on-target specificity. T cell persistence is a function of memory status, exhaustion, and proliferative capacity. In clinical and murine models, ACT of central and effector memory cells are superior to terminal effector T cells (32, 47). In addition, expression and engagement of exhaustion immune checkpoints prevent anti-tumor T cell activation and subsequent proliferation *in vivo.* Our expanded TAg specific T cells lack terminal effector phenotypic markers and infrequently express canonical immune checkpoints, demonstrating a favorable persistence phenotype that is suitable for ACT. Furthermore, the expanded MCPyV TAg T cells are polyfunctional for Th1 effector cytokines as well as IL-2. IL-2 is a key cytokine associated with early polyfunctional memory T cells and naïve T cells.

In our work, we successfully generated CD4^+^ T cells specific for MCPyV TAg and little response in the CD8^+^ compartment, possibly due to inherent properties of our system. This preference for CD4^+^ T cells may be due to the 15mer length of the peptide libraries, however this peptide library format has readily produced antigen-specific CD8^+^ T cells for other viral targets, such as adenovirus and Epstein-Barr virus (48). Shorter peptide libraries may efficiently stimulate some CD8^+^ restricted epitopes that longer peptides fail to induce (49). It is therefore possible that cryptic HLA class I epitopes exist in the T cell repertoire of heathy individuals. Electroporated APCs and several rounds of stimulation produced MCPyV TAg-specific CD8^+^ T cells from healthy donors (50). However, several studies using HLA class I tetramers and multimers also failed to isolate CD8^+^ MCPyV TAg restricted epitopes from healthy donors, suggesting CD8^+^ TAg specific T cells may be low frequency in the healthy donor T cell repertoire (12, 18–20).

Clinical implementation of MCPyV directed ACT could be combined with additional immunotherapies to tailor the needs of each patient. In this proof of concept study, we used blood from a diverse set of healthy donors to produce T cells *ex vivo* that are specific to antigens present in all VP-MCC tumors. By selecting HLA-matched donors, MCPyV antigens expressed through the entire class II HLA array could be targeted by the T cell product. Off-the-shelf donor derived MCPyV CD4^+^ T cells sharing at least one HLA allele with the recipient could also be used. The possibility of graft-versus-host-disease remains a concern for allogeneic T cell transplant studies, and so current trials take a conservative approach in lymphodepletion preconditioning regimens, dosage, and initial T cell selection (51–53). However, this approach could also be adapted for autologous material from VP-MCC patients, who often maintain a higher MCPyV antigenic burden (54). Potency of these products targeting TAg VP-MCC could be further enhanced by including CD8^+^ T cells with alternative tumor specificities such as MAGE-A3, survivin, and SPA17 expressed by VP-MCC (55–57). Although MCPyV TAg cells minimally express the exhaustion markers PD-1 and TIM3 after production, co-administration with ICIs could potentially improve potency and persistence of the adoptively transferred cells.

In conclusion, we have discovered that heathy donors frequently have circulating CD4^+^ T cells specific for MCPyV TAg, that these cells are present across most HLA types, and that they can be efficiently expanded using peptide libraries under enhanced cytokine culture conditions. This study identifies a practical strategy for treating patients with adoptively transferred TAg specific CD4^+^ T cells for immunotherapy of VP-MCC.

## Data Availability Statement

The original contributions presented in the study are included in the article/**Supplementary Material**. Further inquiries can be directed to the corresponding authors.

## Ethics Statement

The studies involving human participants were reviewed and approved by National Institutes of Health Institutional Review Board. Written informed consent to participate in this study was provided by the participants’ legal guardian/next of kin.

## Author Contributions

PM, IB, and JB conceived the idea for the study. SD, MC, and SW planned and performed the experiments. SD analyzed the data and drafted the manuscript. IB and JB supervised the research. All authors contributed to the article and approved the submitted version.

## Funding

Support for this project was provided by the National Heart, Lung, and Blood Institute intramural research program.

## Conflict of Interest

The authors declare that the research was conducted in the absence of any commercial or financial relationships that could be construed as a potential conflict of interest.
