# Supplementary material for: Robust Production of Merkel Cell Polyomavirus Oncogene Specific T Cells From Healthy Donors for Adoptive Transfer
Source: Front Immunol. 2020 Dec 9;11:592721. doi: 10.3389/fimmu.2020.592721 (PMC7756016; doi:10.3389/fimmu.2020.592721)
Supplement: Supplementary file 1 [file DataSheet_1.docx]

Robust production of Merkel cell polyomavirus oncogene specific

T cells from healthy donors for adoptive transfer

Sarah I. Davies, John Barrett, Susan Wong, Mark Jesse Chang,

Pawel J. Muranski, and Isaac Brownell

# **Supplementary Materials and Methods**

## **Tables of flow cytometry antibodies**

| **Intracellular activation** | |  |  | |  | |  |
| --- | --- | --- | --- | --- | --- | --- | --- |
| **Target** | **Clone** | **Fluorochrome** | | **Company** | | **Cat. No.** | |
| CD14 | TuK4 | Pacific blue | | Invitrogen | | MHCD1428 | |
| CD19 | SJ25-C | Pacific blue | | Invitrogen | | MHCD1928 | |
| CD3 | OKT3 | BV605 | | BioLegend | | 317322 | |
| CD4 | OKT4 | BV510 | | BioLegend | | 317444 | |
| CD8 | SK1 | APC-H7 | | BD Biosciences | | 560179 | |
| Granzyme B  (GZMB) | GB11 | AF 647 | | BioLegend | | 515406 | |
| IFN-y | 4S.B3 | PE | | eBioScience | | 12-7319-42 | |
| IL-2 | MQ1-12H12 | Alexa Fluor® 488 | | BioLegend | | 500314 | |
| PD-1 | EH12.2H7 | BV786 | | BioLegend | | 329930 | |
| TNFα | MAb11 | PE-Cy7 | | eBioScience | | 25-7349-41 | |

| **Multi-cytokine panel** | |  |  |  |
| --- | --- | --- | --- | --- |
| **Target** | **Clone** | **Fluorochrome** | **Company** | **Cat. No.** |
| CD154 | 24-31 | PE-Cy5 | BioLegend | 310808 |
| CD3 | OKT3 | BV605 | BioLegend | 317322 |
| CD4 | OKT4 | BV510 | BioLegend | 317444 |
| IFNG | 4S.B3 | PE | eBioScience | 12-7319-42 |
| IL-10 | 501411 | Alexa Fluor® 488 | BioLegend | JES3-9D7 |
| IL-17A | BL168 | Alexa Fluor®700 | BioLegend | 512318 |
| IL-22 | 2G12A41 | APC | BioLegend | 366705 |
| IL-4 | MP4-25D2 | APC-H7 | BioLegend | 500833 |
| TNFα | MAb11 | PE-Cy7 | eBioScience | 25-7349-41 |

| **Transcription factor panel** | |  |  |  |
| --- | --- | --- | --- | --- |
| **Target** | **Clone** | **Fluorochrome** | **Company** | **Cat. No.** |
| CD154 | 24-31 | PE-Cy7 | eBioscience | 25-1548-42 |
| CD3 | OKT3 | BV605 | BioLegend | 317322 |
| CD4 | SK3 | Alexa Fluor®700 | BioLegend | 344822 |
| CD8 | RPA-TA | PE | BioLegend | 301008 |
| FOXP3 | 236A/E7 | APC | eBioscience | 17-4777-47 |
| PD-1 | EH12.2H7 | BV786 | BioLegend | 329930 |
| T-BET | 4B10 | Alexa Fluor® 488 | BioLegend | 644830 |

| **CD137 panel** |  |  |  |  |
| --- | --- | --- | --- | --- |
| **Target** | **Clone** | **Fluorochrome** | **Company** | **Cat. No.** |
| CD137 (4-1BB) | 4B4-1 | APC | BioLegend | 309810 |
| CD3 | OKT3 | BV605 | BioLegend | 317322 |
| CD4 | OKT4 | BV510 | BioLegend | 317444 |
| CD8 | SK1 | APC-Cy7 | BD Biosciences | 560179 |
| PD-1 | EH12.2H7 | BV786 | BioLegend | 329930 |

| **Memory marker panel** | |  |  |  |
| --- | --- | --- | --- | --- |
| **Target** | **Clone** | **Fluorochrome** | **Company** | **Cat. No.** |
| CCR7 | 150503 | Alexa Fluor®700 | BD Pharmingen | 561143 |
| CD127 | A01905 | BV650 | BioLegend | 351359 |
| CD27 | 0323 | PE-Cy5 | eBioscience | 15-0279-42 |
| CD3 | OKT3 | BV605 | BioLegend | 317322 |
| CD4 | OKT4 | BV510 | BioLegend | 317444 |
| CD45RA | HI100 | PE | BD Pharmingen | 555489 |
| CD45RO | UCHL1 | APC-Cy7 | BioLegend | 304228 |
| CD57 | HNK-1 | APC | BioLegend | 359610 |
| CD62L | DREG-56 | FITC | BD Pharmingen | 555543 |
| CD8 | RPA-T8 | BV570 | BioLegend | 301038 |
| CD95 | DX2 | PE-Cy7 | BioLegend | 305622 |
| PD-1 | EH12.2H7 | BV786 | BioLegend | 329930 |

| **Exhaustion marker panel** | |  |  |  |
| --- | --- | --- | --- | --- |
| **Target** | **Clone** | **Fluorochrome** | **Company** | **Cat. No.** |
| CD137 (4-1BB) | 4B4-1 | PE-Cy5 | BioLegend | 309808 |
| CD3 | OKT3 | BV605 | BioLegend | 317322 |
| CD4 | OKT4 | BV510 | BioLegend | 317444 |
| CD8 | SK1 | APC-H7 | BD Biosciences | 560179 |
| LAG3 | 3DS223H | APC | eBioscience | 17-2239-42 |
| PD-1 | EH12.2H7 | BV786 | BioLegend | 329930 |
| TIM3 | F38-2E2 | FITC | BioLegend | 345022 |

**Gating strategies of flow panels**

**Intracellular activation**


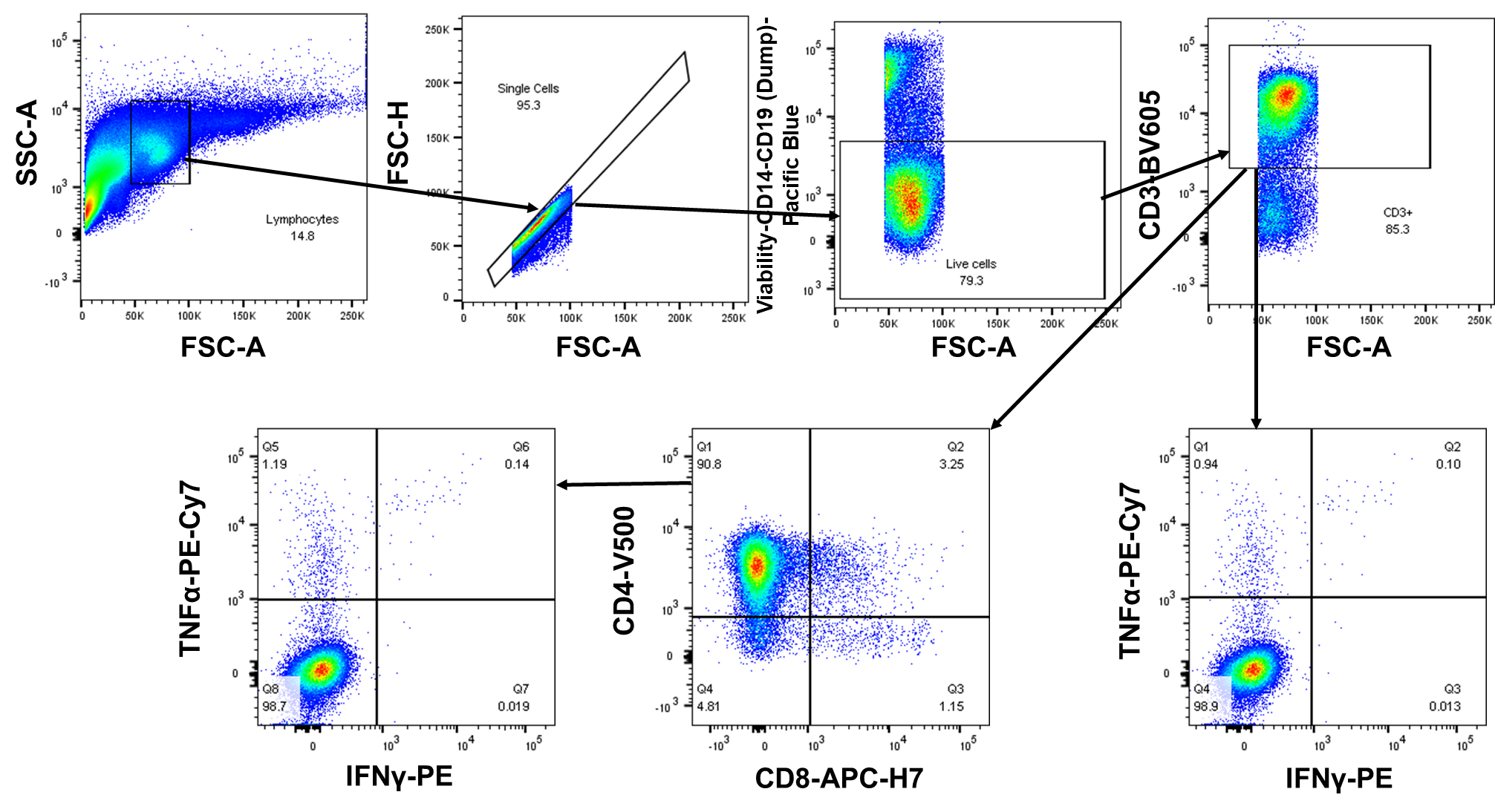


**Intracellular activation-polyfunctionality SPICE plot gating**


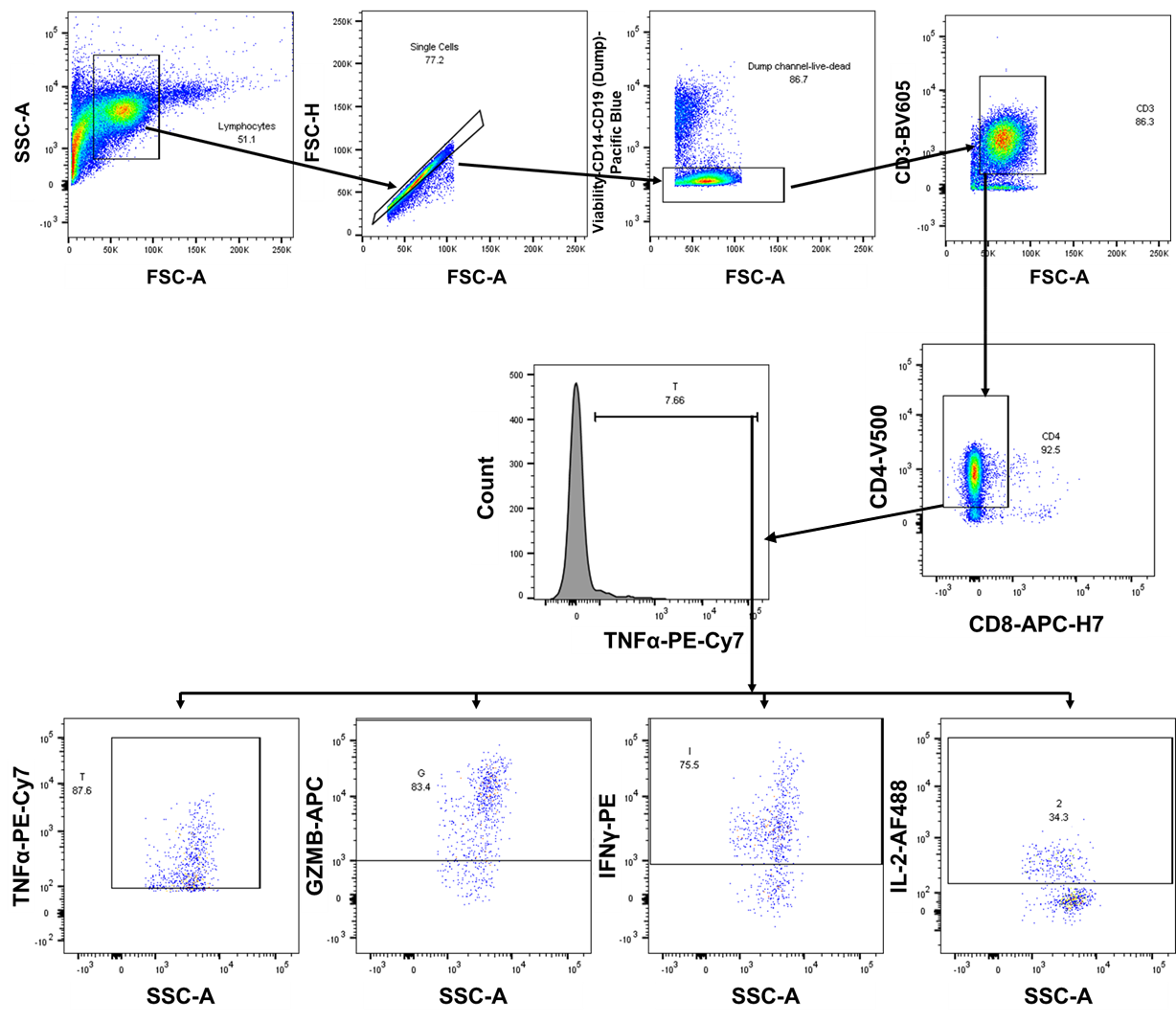


**Multi-cytokine panel**


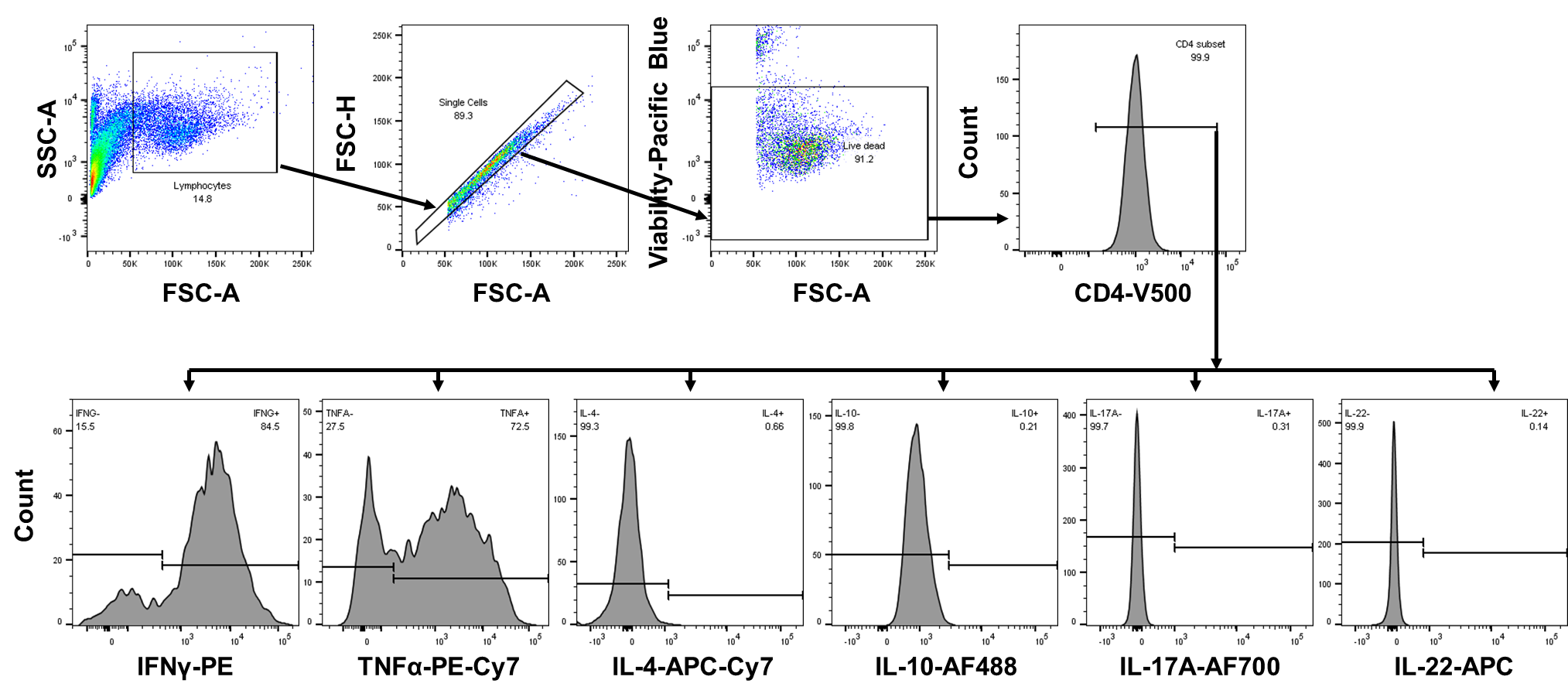


**Transcription factor panel**


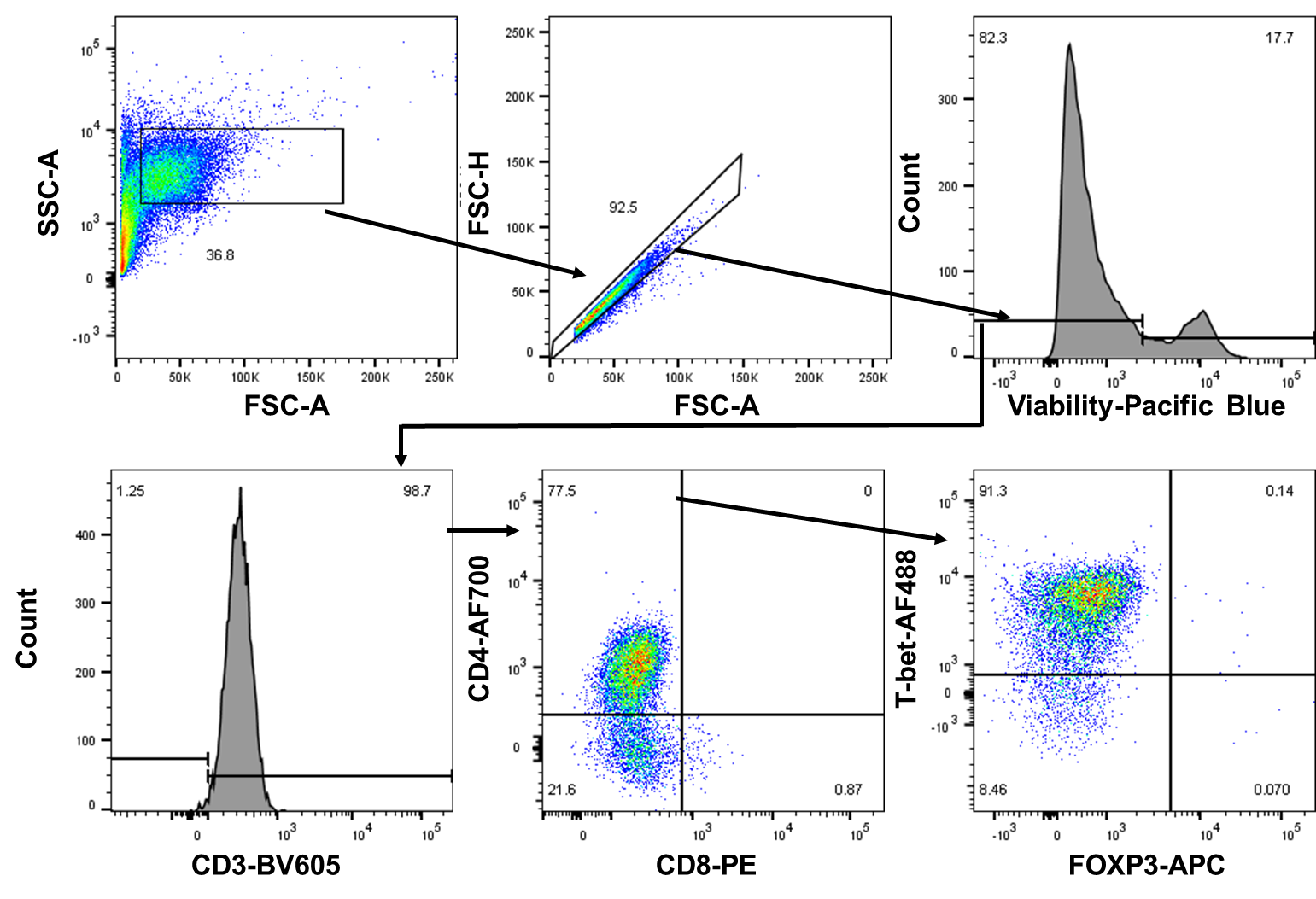


**CD137 panel**

**
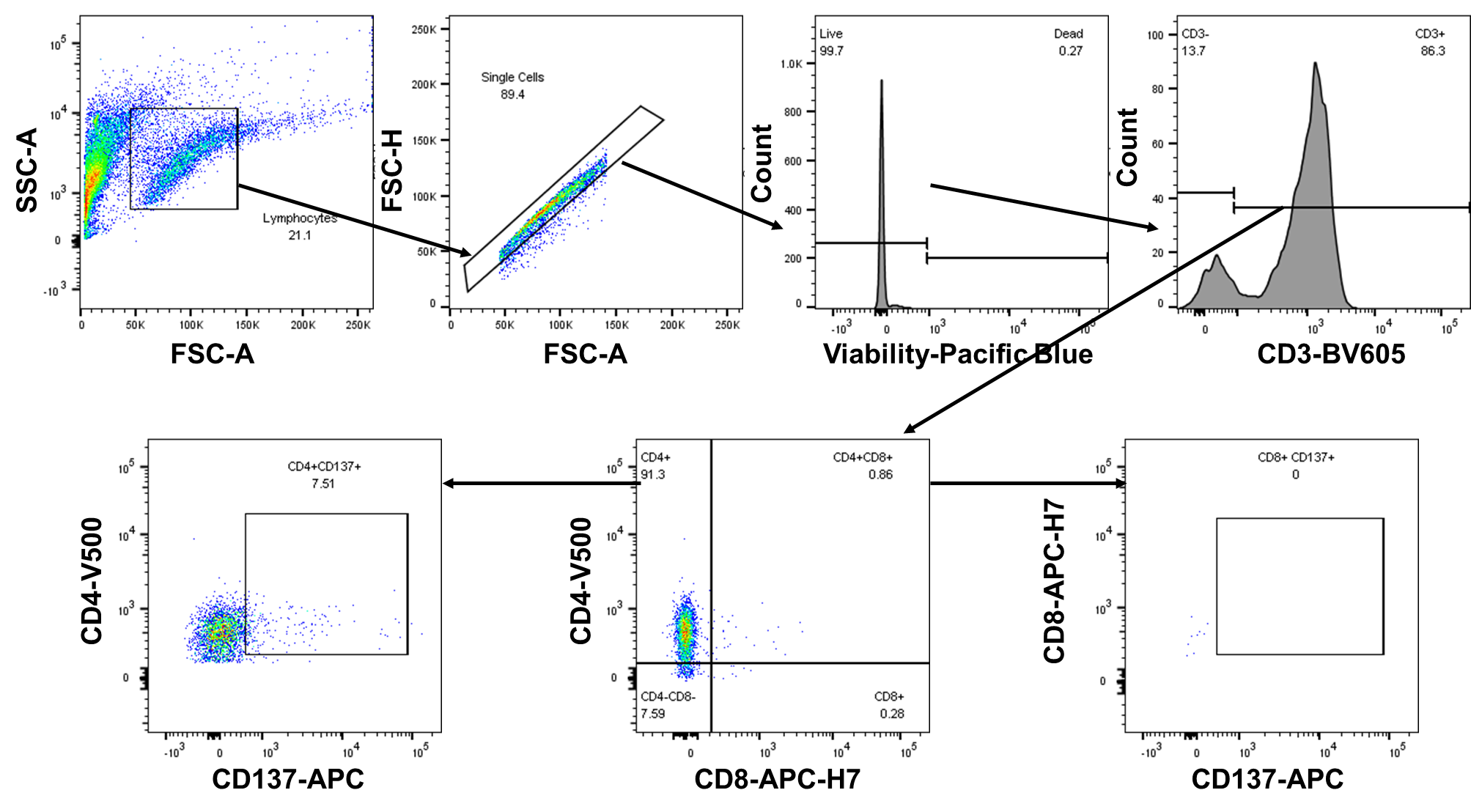
**

**Memory marker panel**

**
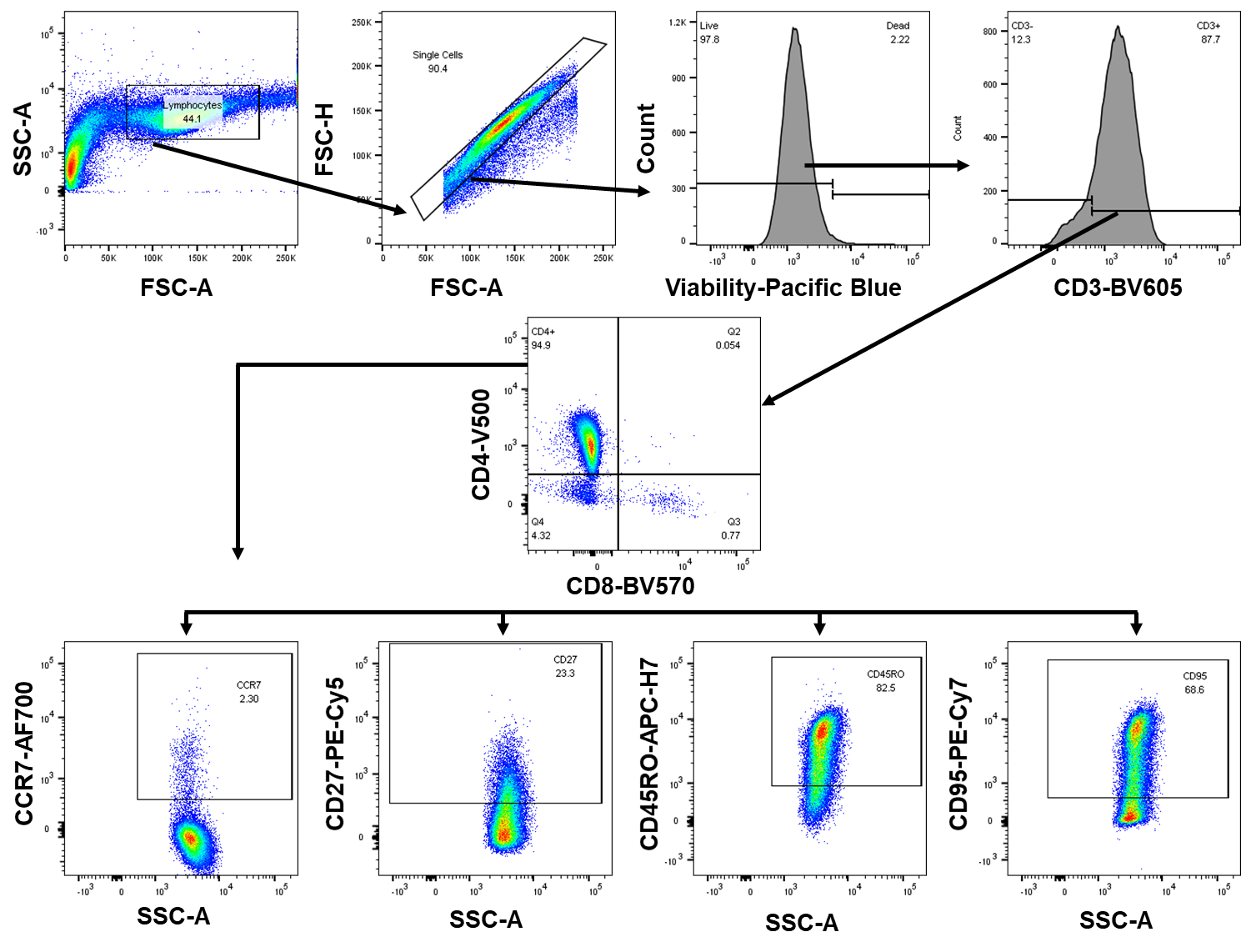
**

**Exhaustion marker panel**

**
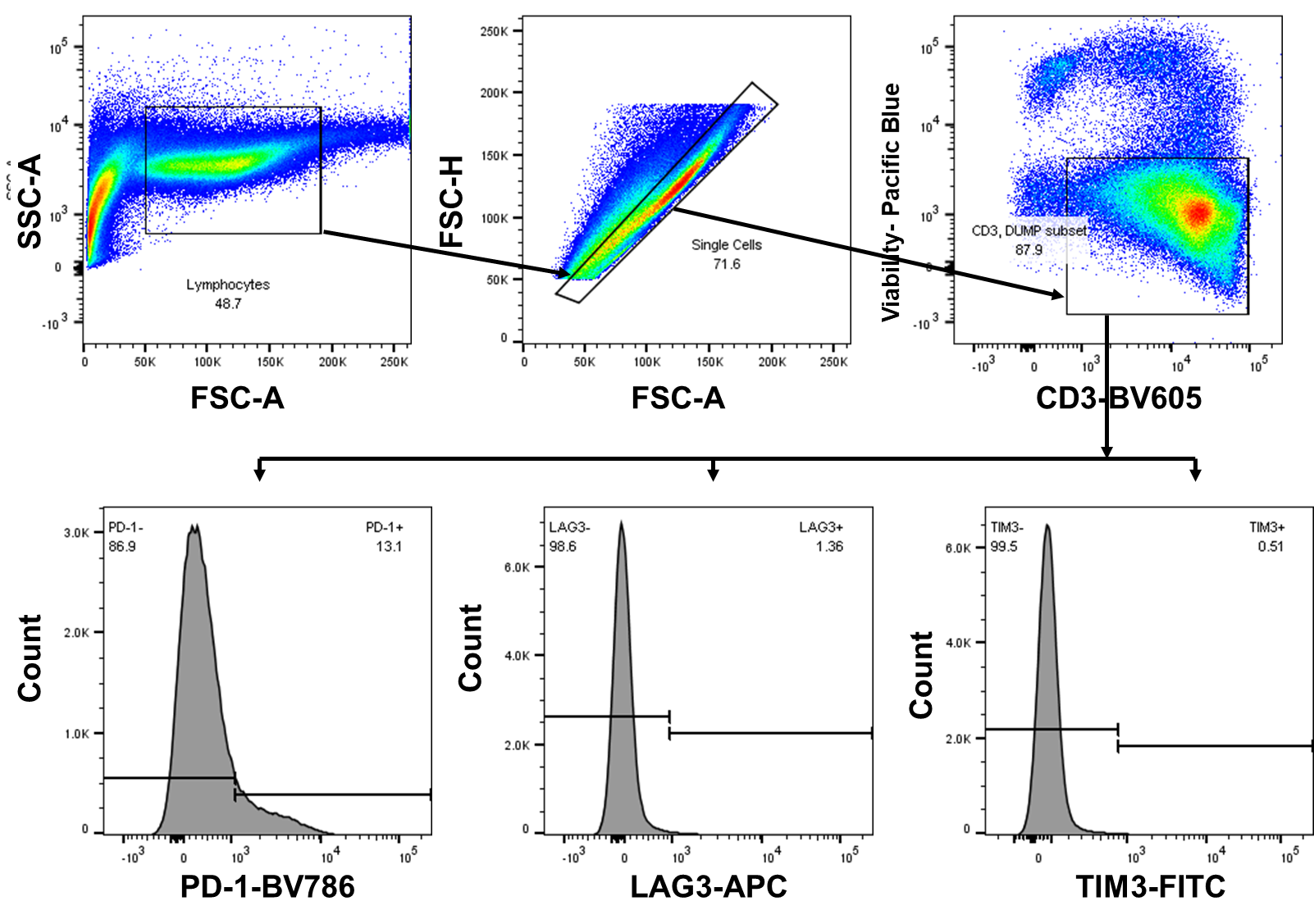
**

## **Sequence of construct and primers for the transduction experiment**

Plasmid backbone pLV-CAG-puro sequence is proprietary and can be purchased by Cellomics (Cat no. CLVP-109-200UL). The ST, LTT, MAGE-A3, and GFP codon optimized sequences were cloned into the EcoR1 /XmaI or EcoRI/BamHI restriction sites and confirmed by PCR.

| **Sequence name** | **Insert sequence** |
| --- | --- |
| **MCPyV ST** | GAATTCGCCACCATGGATCTGGTGCTGAACCGCAAAGAACGCGAAGCGCTGTGCAAACTGCTGGAAATTGCGCCGAACTGCTATGGCAACATTCCGCTGATGAAAGCGGCGTTTAAACGCAGCTGCCTGAAACATCATCCGGATAAAGGCGGCAACCCGGTGATTATGATGGAACTGAACACCCTGTGGAGCAAATTTCAGCAGAACATTCATAAACTGCGCAGCGATTTTAGCATGTTTGATGAAGTGAGCACCAAATTTCCGTGGGAAGAATATGGCACCCTGAAAGATTATATGCAGAGCGGCTATAACGCGCGCTTTTGCCGCGGCCCGGGCTGCATGCTGAAACAGCTGCGCGATAGCAAATGCGCGTGCATTAGCTGCAAACTGAGCCGCCAGCATTGCAGCCTGAAAACCCTGAAACAGAAAAACTGCCTGACCTGGGGCGAATGCTTTTGCTATCAGTGCTTTATTCTGTGGTTTGGCTTTCCGCCGACCTGGGAAAGCTTTGATTGGTGGCAGAAAACCCTGGAAGAAACCGATTATTGCCTGCTGCATCTGCATCTGTTTTGAGGATCC |
| **MCPyV LTT** | GAATTCGCCACCATGGATTTAGTCCTAAATAGGAAAGAAAGAGAGGCTCTCTGCAAGCTTTTAGAGATTGCTCCTAATTGTTATGGCAACATCCCTCTGATGAAAGCTGCTTTCAAAAGAAGCTGCTTAAAGCATCACCCTGATAAAGGGGGAAATCCTGTTATAATGATGGAATTGAACACCCTTTGGAGCAAATTCCAGCAAAATATCCACAAGCTCAGAAGTGACTTCTCTATGTTTGATGAGGTTGACGAGGCCCCTATATATGGGACCACTAAATTCAAAGAATGGTGGAGATCAGGAGGATTCAGCTTCGGGAAGGCATACGAATATGGGCCCAATCCACACGGGACCAACTCAAGATCCAGAAAGCCTTCCTCCAATGCATCCAGGGGAGCCCCCAGTGGAAGCTCACCACCCCACAGCCAGAGCTCTTCCTCTGGGTATGGGTCCTTCTCAGCGTCCCAGGCTTCAGACTCCCAGTCCAGAGGACCCGATATACCTCCCGAACACCATGAGGAACCCACCTCATCCTCTGGATcCAGTAGCAGAGAGGAGACCACCAATTCAGGAAGAGAATCcAGCACACCCAATGGAACcAGTGTACCTAGAAATTCTTCCAGAACGGATGGCACCTGGGAGGATCTCTTcTGCGATGAATCACTTTCCTCCCCTGAGCCTCCCTCGTCCTCTGAGGAGCCTGAGGAGCCCCCCTCCTCAAGAAGCTCGCCCCGGTGACCCGGG |
| **MAGE-A3** | GAATTCGCCACCATGCCCCTTGAGCAGCGCTCACAGCATTGTAAGCCCGAGGAGGGTCTGGAGGCACGAGGTGAAGCCCTCGGTCTCGTAGGTGCTCAGGCCCCTGCAACAGAGGAGCAAGAGGCTGCCTCATCTAGCAGCACCCTTGTGGAGGTCACATTGGGTGAGGTGCCAGCTGCCGAAAGCCCTGATCCCCCGCAGAGTCCTCAAGGTGCTTCCTCTCTCCCTACTACCATGAATTATCCACTCTGGAGCCAGAGCTATGAAGATTCCTCCAATCAGGAAGAAGAGGGCCCTTCTACTTTTCCGGACCTGGAGTCAGAGTTTCAGGCAGCCCTGTCTCGCAAGGTTGCAGAACTTGTGCATTTCCTCCTCCTTAAGTACCGCGCACGCGAACCAGTGACGAAAGCCGAAATGTTGGGCTCTGTGGTGGGTAATTGGCAGTACTTCTTCCCTGTGATATTCTCCAAGGCGAGCAGCTCTCTGCAGCTGGTTTTTGGGATCGAACTCATGGAAGTTGACCCCATTGGACACCTCTACATATTCGCCACGTGTCTCGGACTCTCCTATGACGGGCTCCTGGGTGATAATCAGATTATGCCAAAGGCAGGGCTGCTTATAATTGTACTGGCCATCATTGCCCGGGAAGGGGATTGTGCCCCTGAGGAAAAGATATGGGAGGAGCTTAGCGTCCTGGAAGTGTTCGAGGGGCGGGAGGACTCTATACTCGGTGATCCTAAAAAGCTGCTGACTCAACATTTTGTGCAAGAGAATTACCTGGAGTACCGCCAGGTGCCAGGAAGTGACCCAGCGTGCTACGAGTTTCTCTGGGGACCTAGAGCGCTTGTGGAAACATCTTATGTGAAAGTGTTGCACCACATGGTGAAAATCTCCGGAGGCCCTCACATATCATACCCACCTCTCCACGAGTGGGTGCTGCGAGAAGGGGAGGAGTGAGGATCC |

Expression of codon optimized T antigen constructs in transduced moDCs were determined by RT PCR. MoDCs were harvested two days post transduction for RNA extraction using the Qiagen RNeasy kit. Reverse transcription and subsequent PCR were conducted using the SuperScript III kit (Invitrogen 18080051) and HiFi Hot Start Taq (Kapa Biosystems; KK2601) respectively. Cells transduced with GFP constructs and non-template controls were used as a negative controls. Amplicons were visualized on a 1% agarose gel with ethidium bromide. Thermocycler conditions are as follows:

95C 3min

30x cycles

98C 20sec

55C 15sec

72C 30sec

72C 1min

4C hold

| **Primer name** | **Sequence** | **Expected amplicon length** |
| --- | --- | --- |
| ST forward | GATCTGGTGCTGAACCGCAAAG | 545 |
| ST reverse | CAGATGCAGCAGGCAATAATCG |  |
| LTT forward | GATGGAATTGAACACCCTTTGG | 564 |
| LTT reverse | GGGCGAGCTTCTTGAGGAGG |  |
| MAGE A3 forward | TTGTAAGCCCGAGGAGGGTC | 920 |
| MAGE A3 reverse | CTTCTCGCAGCACCCACTC |  |

ST and LTT proteins are rapidly degraded when transduced individually in primary cells, and insufficient construct protein was detected by western blot using both CM2B4 or 2T2 antibodies (Sigma Aldrich MABF2044 and MABF2316-100UL).

**List of peptide library sequences**

**MCPyV Common T region (Neobiolabs)**

MDLVLNRKEREALCK

LNRKEREALCKLLEI

EREALCKLLEIAPNC

LCKLLEIAPNCYGNI

LEIAPNCYGNIPLMK

PNCYGNIPLMKAAFK

GNIPLMKAAFKRSCL

LMKAAFKRSCLKHHP

AFKRSCLKHHPDKGG

SCLKHHPDKGGNPVI

HHPDKGGNPVIMMEL

KGGNPVIMMELNTLW

PVIMMELNTLWSKFQ

MELNTLWSKFQQNIH

TLWSKFQQNIHKLRS

KFQQNIHKLRSDFSM

NIHKLRSDFSMFDEV

**MCPyV ST unique region (Neobiolabs)**

STKFPWEEYGTLKDY

PWEEYGTLKDYMQSG

YGTLKDYMQSGYNAR

KDYMQSGYNARFCRG

QSGYNARFCRGPGCM

NARFCRGPGCMLKQL

CRGPGCMLKQLRDSK

GCMLKQLRDSKCACI

KQLRDSKCACISCKL

DSKCACISCKLSRQH

ACISCKLSRQHCSLK

CKLSRQHCSLKTLKQ

RQHCSLKTLKQKNCL

SLKTLKQKNCLTWGE

LKQKNCLTWGECFCY

NCLTWGECFCYQCFI

WGECFCYQCFILWFG

FCYQCFILWFGFPPT

CFILWFGFPPTWESF

WFGFPPTWESFDWWQ

PPTWESFDWWQKTLE

ESFDWWQKTLEETDY

WWQKTLEETDYCLLH

**MCPyV LT exon 2 (Neobiolabs)**

DEAPIYGTTKFKEWW

IYGTTKFKEWWRSGG

TKFKEWWRSGGFSFG

EWWRSGGFSFGKAYE

SGGFSFGKAYEYGPN

SFGKAYEYGPNPHGT

AYEYGPNPHGTNSRS

GPNPHGTNSRSRKPS

HGTNSRSRKPSSNAS

SRSRKPSSNASRGAP

KPSSNASRGAPSGSS

NASRGAPSGSSPPHS

GAPSGSSPPHSQSSS

GSSPPHSQSSSSGYG

PHSQSSSSGYGSFSA

SSSSGYGSFSASQAS

GYGSFSASQASDSQS

FSASQASDSQSRGPD

QASDSQSRGPDIPPE

SQSRGPDIPPEHHEE

GPDIPPEHHEEPTSS

PPEHHEEPTSSSGSS

HEEPTSSSGSSSREE

TSSSGSSSREETTNS

GSSSREETTNSGRES

REETTNSGRESSTPN

TNSGRESSTPNGTSV

RESSTPNGTSVPRNS

TPNGTSVPRNSSRTD

TSVPRNSSRTDGTWE

RNSSRTDGTWEDLFC

RTDGTWEDLFCDESL

TWEDLFCDESLSSPE

LFCDESLSSPEPPSS

ESLSSPEPPSSSEEP

SPEPPSSSEEPEEPP

PSSSEEPEEPPSSRS

EEPEEPPSSRSSPRQ

EPPSSRSSPRQPPSS

**WT1 (JPT peptide technologies; Product Code: PM-WT1)**

MGSDVRDLNALLPAV

VRDLNALLPAVPSLG

NALLPAVPSLGGGGG

PAVPSLGGGGGCALP

SLGGGGGCALPVSGA

GGGCALPVSGAAQWA

ALPVSGAAQWAPVLD

SGAAQWAPVLDFAPP

QWAPVLDFAPPGASA

VLDFAPPGASAYGSL

APPGASAYGSLGGPA

ASAYGSLGGPAPPPA

GSLGGPAPPPAPPPP

GPAPPPAPPPPPPPP

PPAPPPPPPPPPHSF

PPPPPPPPHSFIKQE

PPPPHSFIKQEPSWG

HSFIKQEPSWGGAEP

KQEPSWGGAEPHEEQ

SWGGAEPHEEQCLSA

AEPHEEQCLSAFTVH

EEQCLSAFTVHFSGQ

LSAFTVHFSGQFTGT

TVHFSGQFTGTAGAC

SGQFTGTAGACRYGP

TGTAGACRYGPFGPP

GACRYGPFGPPPPSQ

YGPFGPPPPSQASSG

GPPPPSQASSGQARM

PSQASSGQARMFPNA

SSGQARMFPNAPYLP

ARMFPNAPYLPSCLE

PNAPYLPSCLESQPA

YLPSCLESQPAIRNQ

CLESQPAIRNQGYST

QPAIRNQGYSTVTFD

RNQGYSTVTFDGTPS

YSTVTFDGTPSYGHT

TFDGTPSYGHTPSHH

TPSYGHTPSHHAAQF

GHTPSHHAAQFPNHS

SHHAAQFPNHSFKHE

AQFPNHSFKHEDPMG

NHSFKHEDPMGQQGS

KHEDPMGQQGSLGEQ

PMGQQGSLGEQQYSV

QGSLGEQQYSVPPPV

GEQQYSVPPPVYGCH

YSVPPPVYGCHTPTD

PPVYGCHTPTDSCTG

GCHTPTDSCTGSQAL

PTDSCTGSQALLLRT

CTGSQALLLRTPYSS

QALLLRTPYSSDNLY

LRTPYSSDNLYQMTS

YSSDNLYQMTSQLEC

NLYQMTSQLECMTWN

MTSQLECMTWNQMNL

LECMTWNQMNLGATL

TWNQMNLGATLKGVA

MNLGATLKGVAAGSS

ATLKGVAAGSSSSVK

GVAAGSSSSVKWTEG

GSSSSVKWTEGQSNH

SVKWTEGQSNHSTGY

TEGQSNHSTGYESDN

SNHSTGYESDNHTTP

TGYESDNHTTPILCG

SDNHTTPILCGAQYR

TTPILCGAQYRIHTH

LCGAQYRIHTHGVFR

QYRIHTHGVFRGIQD

HTHGVFRGIQDVRRV

VFRGIQDVRRVPGVA

IQDVRRVPGVAPTLV

RRVPGVAPTLVRSAS

GVAPTLVRSASETSE

TLVRSASETSEKRPF

SASETSEKRPFMCAY

TSEKRPFMCAYPGCN

RPFMCAYPGCNKRYF

CAYPGCNKRYFKLSH

GCNKRYFKLSHLQMH

RYFKLSHLQMHSRKH

LSHLQMHSRKHTGEK

QMHSRKHTGEKPYQC

RKHTGEKPYQCDFKD

GEKPYQCDFKDCERR

YQCDFKDCERRFSRS

FKDCERRFSRSDQLK

ERRFSRSDQLKRHQR

SRSDQLKRHQRRHTG

QLKRHQRRHTGVKPF

HQRRHTGVKPFQCKT

HTGVKPFQCKTCQRK

KPFQCKTCQRKFSRS

CKTCQRKFSRSDHLK

QRKFSRSDHLKTHTR

SRSDHLKTHTRTHTG

HLKTHTRTHTGKTSE

HTRTHTGKTSEKPFS

HTGKTSEKPFSCRWP

TSEKPFSCRWPSCQK

PFSCRWPSCQKKFAR

RWPSCQKKFARSDEL

CQKKFARSDELVRHH

FARSDELVRHHNMHQ

DELVRHHNMHQRNMT

RHHNMHQRNMTKLQL

MHQRNMTKLQLAL

**NY-ESO-1 (JPT peptide technologies; Product Code: PM-NYE)**

MQAEGRGTGGSTGDA

GRGTGGSTGDADGPG

GGSTGDADGPGGPGI

GDADGPGGPGIPDGP

GPGGPGIPDGPGGNA

PGIPDGPGGNAGGPG

DGPGGNAGGPGEAGA

GNAGGPGEAGATGGR

GPGEAGATGGRGPRG

AGATGGRGPRGAGAA

GGRGPRGAGAARASG

PRGAGAARASGPGGG

GAARASGPGGGAPRG

ASGPGGGAPRGPHGG

GGGAPRGPHGGAASG

PRGPHGGAASGLNGC

HGGAASGLNGCCRCG

ASGLNGCCRCGARGP

NGCCRCGARGPESRL

RCGARGPESRLLEFY

RGPESRLLEFYLAMP

SRLLEFYLAMPFATP

EFYLAMPFATPMEAE

AMPFATPMEAELARR

ATPMEAELARRSLAQ

EAELARRSLAQDAPP

ARRSLAQDAPPLPVP

LAQDAPPLPVPGVLL

APPLPVPGVLLKEFT

PVPGVLLKEFTVSGN

VLLKEFTVSGNILTI

EFTVSGNILTIRLTA

SGNILTIRLTAADHR

LTIRLTAADHRQLQL

LTAADHRQLQLSISS

DHRQLQLSISSCLQQ

LQLSISSCLQQLSLL

ISSCLQQLSLLMWIT

LQQLSLLMWITQCFL

SLLMWITQCFLPVFL

WITQCFLPVFLAQPP

CFLPVFLAQPPSGQR

VFLAQPPSGQRR
