# Supplementary material for: Robust Production of Merkel Cell Polyomavirus Oncogene Specific T Cells From Healthy Donors for Adoptive Transfer
Source: Front Immunol. 2020 Dec 9;11:592721. doi: 10.3389/fimmu.2020.592721 (PMC7756016; doi:10.3389/fimmu.2020.592721)
Supplement: Supplementary file 2 [file DataSheet_2.docx]

# **Supplementary Figures**

**A**


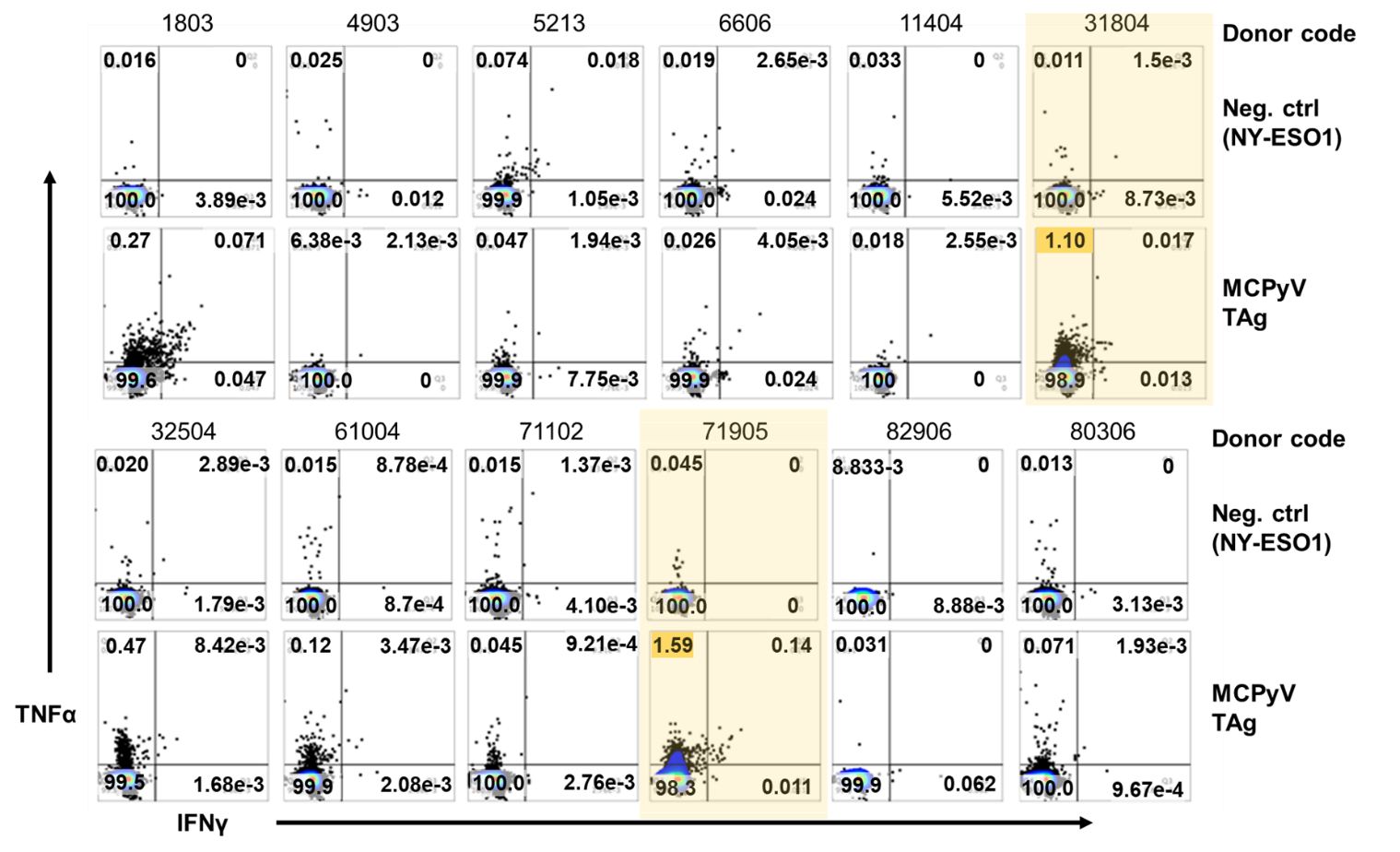


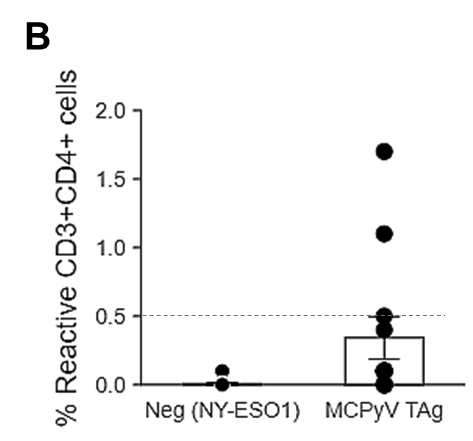


**Supplementary Figure 1.** CD4 enriched T cells expanded in standard medium cytokines IL-2, IL-7, and IL-15 yield low frequencies of MCPyV TAg specific T cells (n=12). T cells were enriched for CD3+CD4+ T cells and stimulated by MCPyV TAg peptide loaded moDCs. After two total rounds of stimulation, cultures were challenged with either MCPyV TAg or an irrelevant peptide library, NY-ESO1. **(A)** Individual plots gated on live CD3+CD4+ cells and reactive donors are highlighted in yellow. **(B)** Summary bar chart. Paired T-test p=0.0557.


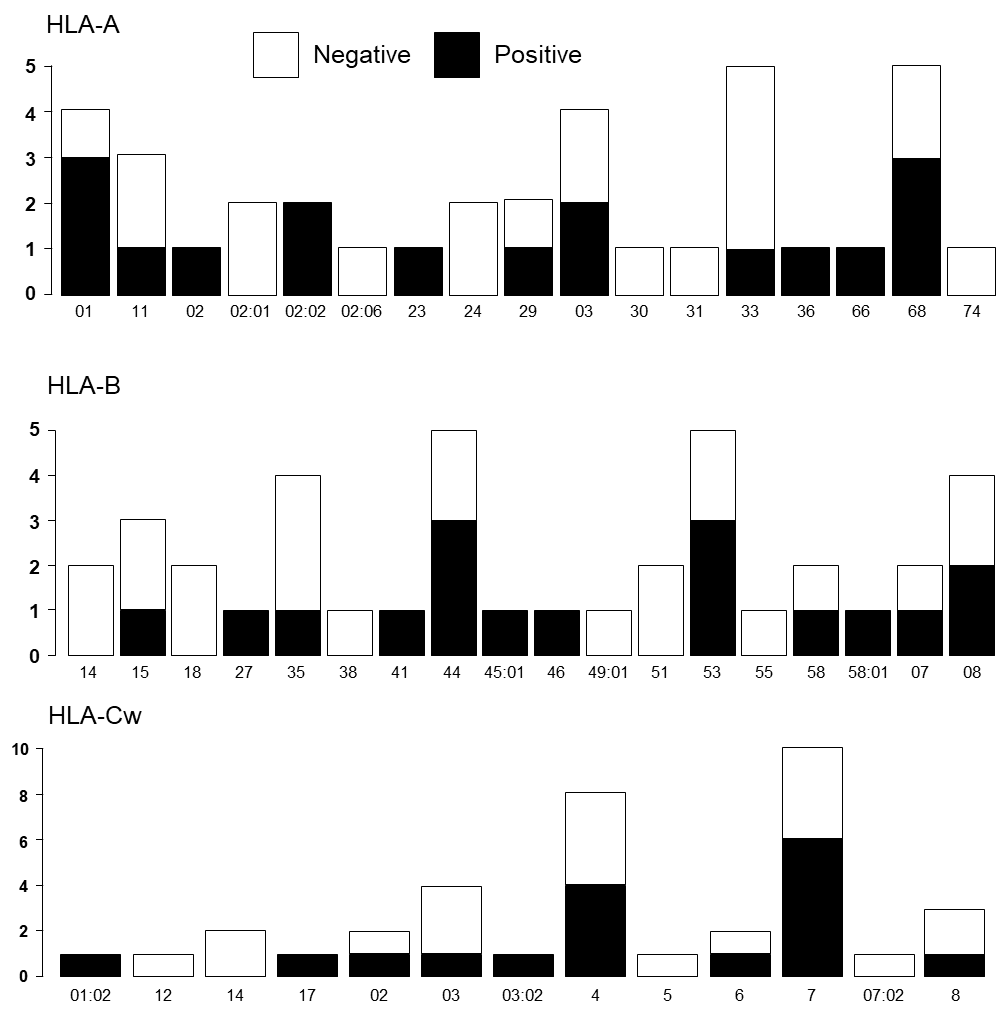


**Supplementary Figure 2.** Distribution of HLA class I alleles of negative (n=11) and positive (n=9) MCPyV TAg responding healthy donors. No bias for HLA alleles by Fisher’s exact test for HLA-A (p=0.5466), B (p=0.7772), or Cw (p=0.8991).


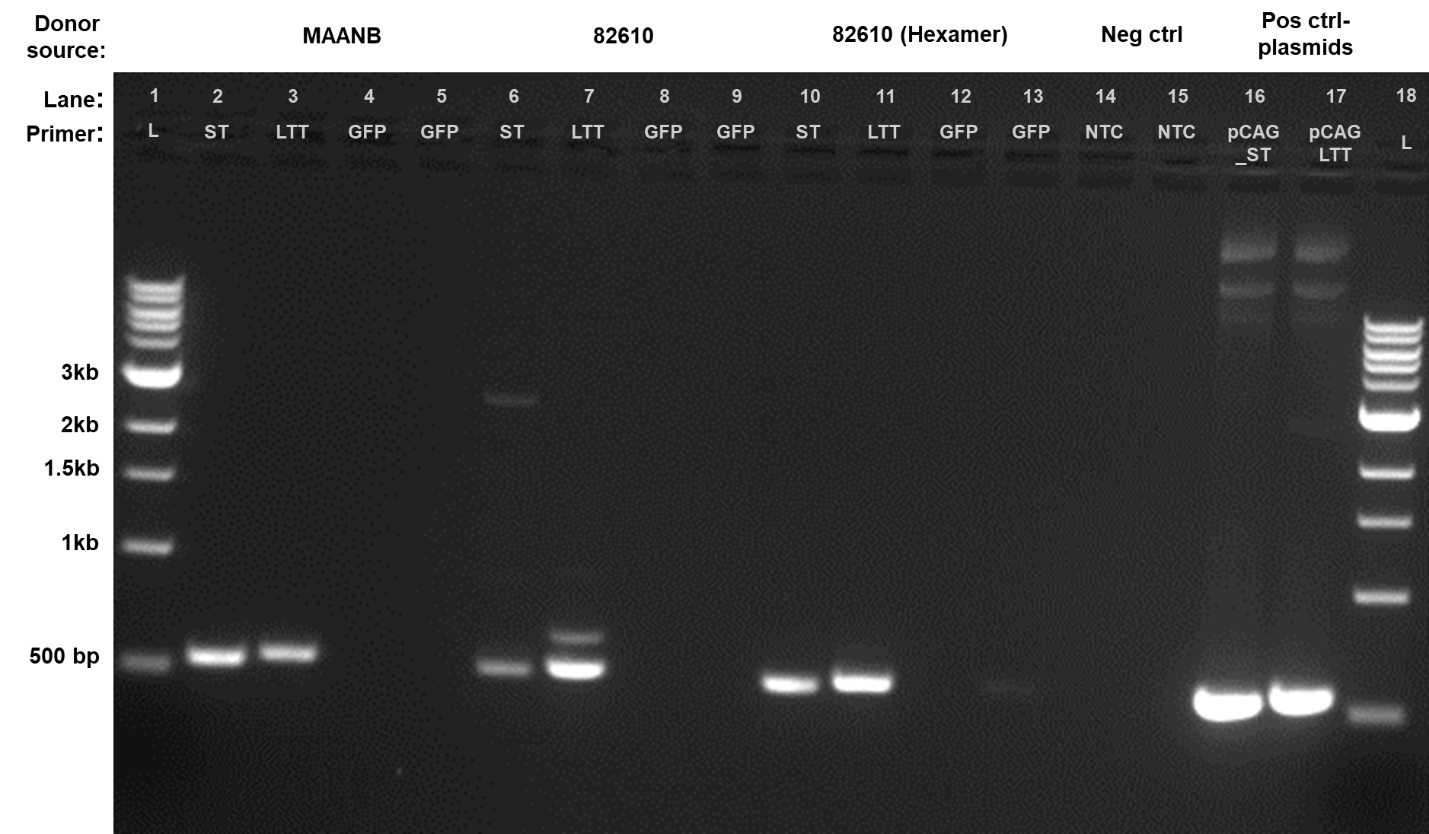


**Supplementary Figure 3.** Transduced moDCs express transduced ST and LTT constructs. Representative gel of transduced moDC lentivirus construct expression of two healthy donors MAANB (lanes 2-5) and 82610 (lanes 6-13). RNA samples were reverse transcribed using oligo(d)T in lanes 2-9 and a random hexamer in lanes 10-13. Non template controls are in lanes for the ST and LTT primer sets are in lane 14 and 15 respectively. Purified plasmids containing ST and LTT respectively were positive controls in lanes 16 and 17 respectively. GFP primers were used an additional negative control. A 1kb ladder is in lanes 1 and 18 (New England Biolabs N0468S). Expected amplicon of the primer sets are 545bp for the ST primer set and 564bp for the LTT primer set. Gel was 1% agarose and DNA was visualized with ethidium bromide.
